# Antibacterial Hydrogel Sheet Dressings Composed of Poly(vinyl alcohol) and Silver Nanoparticles by Electron Beam Irradiation

**DOI:** 10.3390/gels9020080

**Published:** 2023-01-18

**Authors:** Rattanakorn Chiangnoon, Pennapa Karawak, Jarurattana Eamsiri, Sasikarn Nuchdang, Nuatawan Thamrongsiripak, Naruemon Neramitmansook, Siwanut Pummarin, Pimchanok Pimton, Kewalee Nilgumhang, Pimpon Uttayarat

**Affiliations:** 1 Nuclear Technology Research and Development Center, Thailand Institute of Nuclear Technology (Public Organization), Ongkarak, Nakhon Nayok 26120, Thailand; rattanakorn@tint.or.th (R.C.); pennapa@tint.or.th (P.K.); jaruratana@tint.or.th (J.E.); sasikarn@tint.or.th (S.N.); 2 Irradiation Center, Thailand Institute of Nuclear Technology (Public Organization), Ongkarak, Nakhon Nayok 26120, Thailand; nuatawan@tint.or.th (N.T.); naruemon@tint.or.th (N.N.); 3 Department of Biology, School of Science, Walailak University, Nakhon Si Thammarat 80160, Thailand; p.siwanut@gmail.com (S.P.); pimchanok.pi@mail.wu.ac.th (P.P.); 4 Program in Medical Sciences, Faculty of Medicine, Chulalongkorn University, Bangkok 10330, Thailand; 5 Functional Materials and Nanotechnology Center of Excellence, Walailak University, Nakhon Si Thammarat 80160, Thailand; 6 Advanced Engineering and Nuclear Technology Center, Thailand Institute of Nuclear Technology (Public Organization), Ongkarak, Nakhon Nayok 26120, Thailand; kewalee@tint.or.th

**Keywords:** hydrogel sheet dressings, poly(vinyl alcohol), silver nanoparticles, electron beam irradiation, antibacterial

## Abstract

Advanced wound dressings that can deliver potent antibacterial action are still much in need, especially for treating wound infections caused by drug-resistant bacteria. In this research, we utilized electron beam (EB) irradiation to develop antibacterial hydrogel sheet dressings from poly(vinyl alcohol) (PVA) and silver nanoparticles (AgNPs) in a two-step processing and evaluated their bactericidal efficacy, as well as the AgNP release. The effect of the irradiation dose on the swelling, gel fraction, network parameters, and mechanical properties of the hydrogels was first determined to establish the optimal doses for the two-step processing. The prototypic hydrogel sheets were then formed in the first EB irradiation and served as a matrix for the AgNP synthesis by the reduction of the silver nitrate precursors during the second EB irradiation. The diffusion assay showed that the minimal inhibition concentration (MIC) of the AgNP-load hydrogels was 0.25 and 0.5 mg/cm^2^ against *Escherichia coli* and *Staphylococcus aureus*, respectively. At these MIC levels, the released AgNPs increased sharply to near maximum, ~30 and 60 ppm, at 24 h as analyzed by atomic absorption. Therefore, we successfully demonstrated that this two-step processing by EB irradiation provides a convenient platform to fabricate AgNP-loaded hydrogel dressings that can be further developed for wound healing.

## 1. Introduction

Annually, millions of people worldwide suffer from the loss of skin due to the fact of injuries, such as burns, chemical exposure, and diseases [1]. The lack of proper wound treatment can cause serious dehydration and wound infection that becomes life-threatening to the patients and poses major burdens for wound care [2,3]. With drug-resistant bacteria currently threatening the treatment of infected wounds, it is desirable to develop wound dressings that can maintain the appropriate moisture at the wound interface, prevent infection, and be easily removed without causing trauma [4].

Hydrogels have emerged as a new class of dressing materials capable of providing a necessary moist environment for wound healing [2,3,5]. Having three-dimensional (3D) networks of crosslinked hydrophilic polymers that can swell a large amount of water without being disintegrated [6], hydrogels can donate hydration to the wounds in addition to absorbing excess exudate into the structure. The soft consistency and smooth surface of hydrogels also enable them to easily conform to the skin [2] and become nonadherent upon removal [7]. The crosslinked networks of hydrogels can be constructed by physical, chemical, and radiation methods [6,8]. Among these approaches, ionizing radiation from high-energy sources, such as gamma rays or an electron beam (EB), offers many unique advantages, as the crosslinking of polymer chains into a gel can be performed without the use of harmful chemical crosslinkers or initiators at room temperature [9,10]. At the same time, sterilization of the final products can be combined within the processing step [9,10]. Therefore, the radiation processing itself is considered a “green” technology and environmentally friendly. In aqueous polymer systems, the generation of hydroxyl radicals (•OH) and hydrogen atoms (•H) during radiolysis of water by gamma or EB irradiation serve as the key agents that induce intermolecular crosslinking of polymer chains into a permanent 3D network [11].

With the increasing demands for advanced wound dressings to treat infected wounds without the overuse of antibiotics, silver nanoparticles (AgNPs) have gained considerable attention, since they exhibit potency across a broad spectrum of bacteria [12,13]. When bound to proteins of cell membranes, internal organelles, and reaction pathways, AgNPs can disrupt protein function which, in turn, lead to bacterial cell death [14]. A recent study by Liu et al. [3] also showed that AgNPs could serve as an agent for photothermal effects to further enhance bacterial cell death via locally elevated temperature. Incorporation of AgNPs into hydrogels can be performed either by synthesizing AgNPs separately before mixing with hydrogel precursors [3] or by combining the formation of AgNPs within the hydrogel crosslinking process [15]. Using the latter approach, Leawhiran et al. [15] demonstrated that AgNP/gelatin/poly(vinyl alcohol) hydrogels could be processed within a single exposure to gamma irradiation. During irradiation, the hydrated electrons (e^−^_aq_) generated by radiolysis reduced Ag^+^ in the starting AgNO_3_ to Ag^0^ that later aggregated to form AgNPs. However, the mechanical strength of the hydrogels was compromised, as the reduction in AgNPs also decreased the extent of the crosslinked polymer matrix [15].

The two-step processing of AgNP-loaded hydrogels has previously been explored by Eid et al. [16] in which the hydrogels were first formed by radiation-induced crosslinking of poly(vinyl alcohol) (PVA) and polyvinyl pyrrolidone, followed by the addition of silver precursor, which was finally reduced into AgNPs by sodium borohydride. As the resulting AgNP-loaded hydrogels will be interfaced with body tissue, caution and consideration is required concerning any remaining harmful chemicals within hydrogels, as well as the sterilization of final products. Therefore, the ability to produce sterilized AgNP-loaded hydrogels with an environmentally friendly method is advantageous and of great interest.

**Figure 1:**
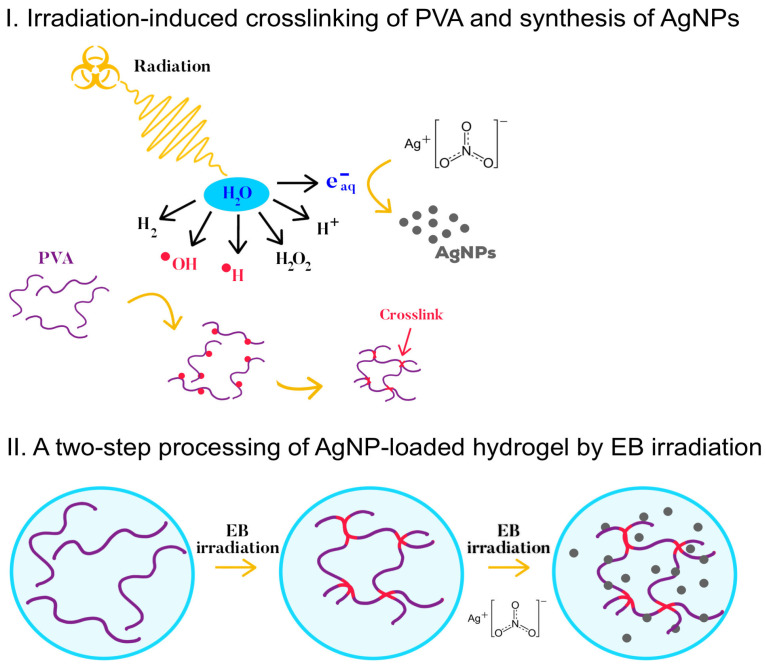
Preparation scheme of antibacterial AgNP-loaded hydrogel sheets by EB irradiation.

In the present study, we demonstrated for the first time the use of EB irradiation to develop antibacterial hydrogel dressings in a two-step processing in which AgNPs were formed by the reduction of silver nitrate (AgNO_3_) precursors during the second irradiation at the sterilization dose (Figure 1). PVA was chosen due to the fact of its biocompatibility, nontoxicity, and water solubility [17]. To elucidate the effect of the irradiation dose on the water absorption, crosslinking, and mechanical properties of the hydrogels, gamma irradiation was first applied to fabricate hydrogel sheet models at doses that ranged from 10 to 80 kGy. The effect of repeated irradiation at a sterilization dose of 25 kGy was then investigated regarding the hydrogels’ swelling and mechanical properties to establish the optimal doses for the two-step processing by EB irradiation. Finally, the antibacterial efficacy, release of AgNPs, and cytocompatibility of the AgNP-loaded hydrogel sheet dressings were evaluated.

## 2. Results and Discussion

### 2.1. Structural Characterization of the Hydrogels

To elucidate the effect of the irradiation dose on the swelling, gel fraction, and crosslinked network parameters of the hydrogels, we first applied gamma irradiation to form hydrogel models from starting PVA solutions at varied doses. Table 1 shows that the gel fraction and EDS of the hydrogels were at opposing trends with the increase in gamma doses. The gel fraction was first measurable (~25%) at a dose of 10 kGy before it increased significantly to 86% when the dose was raised to 25 kGy. At higher doses, the gel fraction further increased to above 90% and remained relatively constant up to the highest dose of 80 kGy. Regarding the swelling capacity of the hydrogels compared to their dried weights, a sharp reduction of the EDS from 2500% to 1300% as the gamma dose was raised from 25 to 40 kGy. A further increase in the doses caused a slight reduction in the EDS to 890% at the highest dose of 80 kGy. The samples formed at 10 kGy were not included in the EDS study due to the fact of their flowable, paste-like appearance that was difficult to handle. Based on these results, the increase in the irradiation dose induced more linking of PVA chains into 3D networks, resulting in the smaller space between the crosslink units to accommodate water molecules.

In addition to the gel fraction and EDS obtained by the experiments, the basic network parameters of the hydrogels, including the average molecular weight between the crosslinks (*M_c_*), crosslink density (*ρ_x_*), and mesh size (*ε*), could also be elucidated by calculation. Using the modified equilibrium swelling theory of Flory and Rehner, assuming an isotropic swelling of the neutral polymer networks in which four polymer chains are joined at one crosslink point [18], *M_c_* can be calculated by the following equation:

(1) $$\frac{1}{M_{c}}=\frac{2}{M_{n}}-\frac{\upsilon /V_{1}[\ln\left(1-V_{2,s}\right)+V_{2,s}+\mu V_{2,s}^{2}}{V_{2,r}[\left(V_{2,s}/V_{2,r}{)}^{1/3}-0.5\left(\frac{V_{2,S}}{V_{2,R}}\right)\right]}$$

where *M_n_* is the number of the average molecular weight (~52,800 based on the manufacturer’s data), υ is the specific volume of PVA (0.788 cm^3^/g), *V*_1_ is the molar volume of water (18 cm^3^/mol), *m* is the Flory–Huggins interaction parameter (0.494 for PVA-water) [19], and *V*_2,_*_r_* and *V*_2,_*_s_* are polymer fractions of hydrogel in a relaxed state and swollen state at equilibrium, respectively. These polymer fractions can be derived from the weights of the original hydrogel, swollen hydrogel, and dried hydrogel, assuming the volume additivity of water and PVA [18,20]. Subsequently, *ρ_x_* and *ε* can be calculated by the following equations [21]:

(2) $$\rho_{x}=\frac{1}{\upsilon M_{c}},$$

(3) $$\varepsilon ={\left(V_{2,s}\right)}^{-\frac{1}{3}}\;{\left[C_{n}\left(\frac{2M_{c}}{M_{r}}\right)\right]}^{\frac{1}{2\;}}l$$

where *C_n_* is the Flory characteristics ratio (8.3 for PVA), *M_r_* is the average molecular weight of repeating unit (44 g/mol for VOH), and *l* is the C-C bond length (1.54 Å) [19].

Table 1 shows that the calculated *M_c_* and *ρ_x_* values are also at opposing trends with an irradiation dose similar to the EDS and gel fraction data. These *M_c_* and *ρ_x_* values obtained in our study were also within the same range as those previously reported in a similar PVA hydrogel system formed by EB irradiation [22]. Following the same trend of *M_c_* with the irradiation doses, the *ε* values also decreased from 358 to 130 Å as the gamma dose increased from 25 to 80 kGy. Based on the calculation of these network parameters, it was clear that as *ρ_x_* increased with the number of crosslink units induced by irradiation, the space or mesh size to accommodate water molecules inside the hydrogel matrix became smaller, resulting in a lowered swelling capacity. Although the values of the gel fraction remained near its maximum at doses of 25–60 kGy, *ρ_x_* continued to increase within this dose range. It may be deduced that despite having most of the polymer chains already joined together by crosslinking at a dose of 25 kGy, more linking of the chains continued to proceed at higher doses. However, at the highest dose of 80 kGy, we observed that our hydrogel sheets became easily torn at the edges during the release from molds. This could be attributed to other modes of radical interaction, such as chain scissions that start to compete with crosslinking [11].

### 2.2. Thermal Properties of the Crosslinked Hydrogels

**Figure 2:**
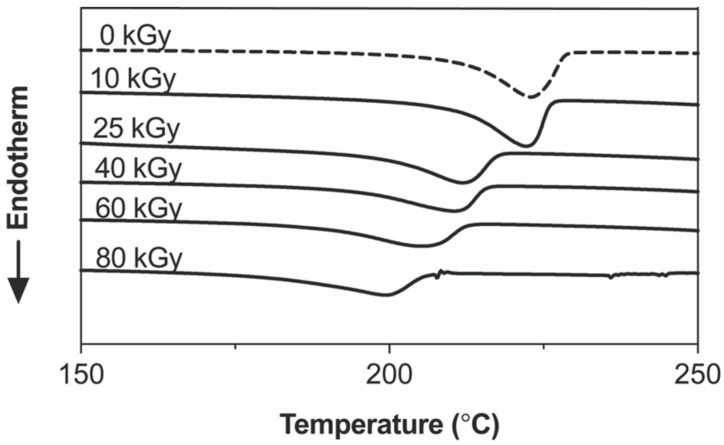
DSC analysis of the crosslinked PVA hydrogel sheets at varied irradiation doses.

The extent of the crosslinked 3D networks inside the hydrogel sheets could also be detected by changes in the thermal properties (Figure 2). A large and sharp endodermic curve that peaked at approximately 227 °C was observed in the nonirradiated samples (0 kGy). After irradiation at increasing doses from 10 to 80 kGy, the melting enthalpies and melting points decreased with broader endodermic peaks, especially at doses greater than 25 kGy. These changes in the thermal properties could be due to the decrease in PVA molecules that were arranged orderly into crystallite domains through hydrogen bonding of the -OH side chains, as they were instead chemically joined into 3D networks. These thermal properties of our crosslinked hydrogels are in agreement with previous studies [23,24] that reported a noticeable drop in the melting enthalpies and melting points of PVA hydrogels crosslinked by irradiation.

### 2.3. Morphology of the Hydrogels

**Figure 3:**
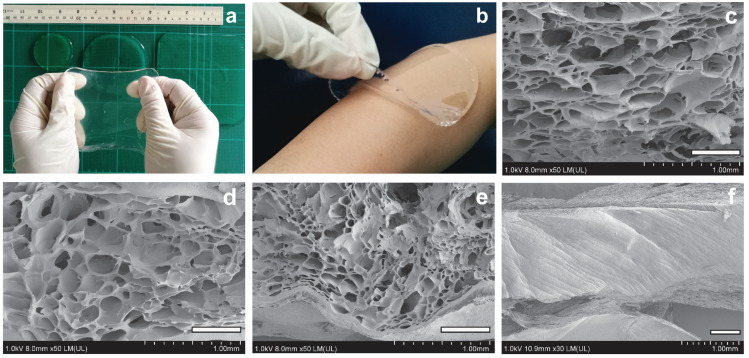
Morphology of the crosslinked PVA hydrogel sheets. Representative digital images of (**a**) stretchable and (**b**) conformal hydrogel sheets fabricated by irradiation at 40 kGy. SEM images showing a porous microstructure inside the hydrogel matrices crosslinked at doses of (**c**) 25, (**d**) 40, and (**e**) 60 kGy compared to the (**f**) as-cast hydrogel sheet. Scale bars are 500 mm in (**c**–**f**).

In our study, gamma doses of 25, 40, and 60 kGy led to the formation of hydrated and pliable hydrogel sheets that could be easily released from the mold without tearing. Figure 3a,b show that the hydrogel sheets were stretchable and conformal over the skin with ease upon removal. Among the three gamma doses, the doses of 40 and 60 kGy resulted in hydrogel sheets that could maintain their expanded, flat shape during handling, while the sheets that formed at 25 kGy appeared floppy and easily rolled or curled from the sides.

To further investigate the microstructure of 3D networks, the hydrogels were cold fractured and visualized by SEM. Irregular pores with sizes that ranged from ~100 to 500 mm were shown to be distributed throughout the hydrogel matrices (Figure 3c–e) in contrast to the as-cast hydrogels (Figure 3f). The size of the pores observed in SEM images was much larger than the average pore size values calculated by the Flory–Rehner modeling approach (Table 1). Such discrepancies have previously been reported in dextran [10,18] and other PVA [25] hydrogel systems in which the actual size of the macropores was underestimated. Although the modeling approach provides a useful description of the crosslinked network structure inside the hydrogels that varied with irradiation doses, the analysis by SEM had to be used in conjunction to obtain an accurate picture of the pore size.

### 2.4. Effect of Repeated Irradiation on the Sterility, Swelling, and Mechanical Properties of the Hydrogel Sheets

As the second irradiation was conducted at 25 kGy, a dose level that was commonly used to sterilize medical devices [15], our test of the sterility confirmed the absence of the microorganism in broths containing bare hydrogel sheets without AgNPs. We then tested if such repeated irradiation would affect the swelling and mechanical properties of the hydrogels. Previously, we demonstrated that both the gel strength and tensile strength of the hydrogel sheets increased with the irradiation doses used to crosslink the hydrogels having the similar 10% (*w*/*v*) PVA system and gamma doses ranging from 25 to 80 kGy [26]. Therefore, based on our current data on swelling, hydrogel sheets that formed at 25 and 40 kGy were selected for the repeated irradiation experiments, as the swelling became saturated at higher doses. The repeatedly irradiated samples were termed 25r and 40r, respectively.

**Figure 4:**
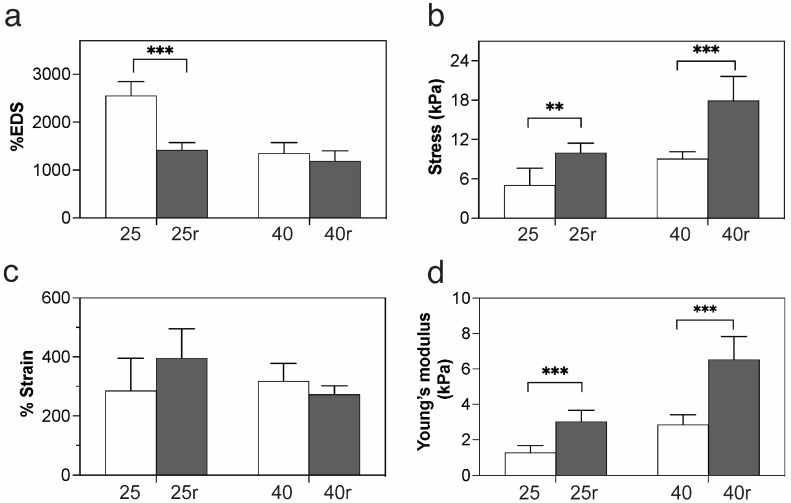
Effect of repeated irradiation on the (**a**) swelling, (**b**) tensile strength, (**c**) elongation at break, and (**d**) Young’s modulus of the hydrogel sheets. Symbols ** and *** correspond to *p*-values of less than 0.01 and 0.001, respectively.

In terms of the swelling, the 25r hydrogels showed a significant reduction in EDS from ~2500 to 1400% after the second exposure to irradiation, whereas the EDS of 40r samples remained relatively the same (Figure 3a). For the tensile properties, both the 25r and 40r samples exhibited a two-fold increase in the tensile strength from ~5 to 10 kPa and ~8 to 18 kPa, respectively. By contrast, the elongation at break showed a slight increase for the 25r and a slight decrease for the 40r samples (Figure 4c). Similar to the tensile strength, the Young’s moduli of both the 25r and 40r samples showed a significant two-fold and three-fold increase, respectively, compared to those without repeated irradiation. Based on these results, the 40 kGy dose was chosen to produce the prototypic hydrogel sheets in the first irradiation step, as their swelling capacity and stretchability were not significantly changed after the second exposure to irradiation at 25 kGy.

### 2.5. Antibacterial Properties of the AgNP-Loaded Hydrogel Sheets

**Figure 5:**
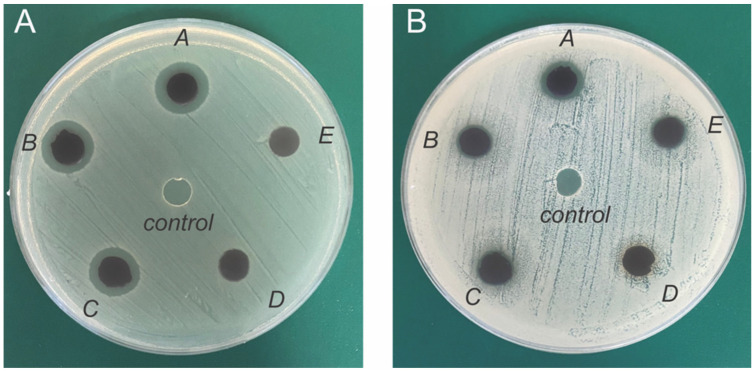
Disc diffusion assay of the AgNP-loaded hydrogels against (**A**) *E. coli* and (**B**) *S. aureus*. Hydrogels A, B, C, D, and E contained AgNPs at 1.0, 0.5, 0.25, 0.125, and 0.0625 mg/cm^2^, respectively. *Control* refers to the hydrogel without AgNPs.

Based on the optimized irradiation doses obtained from the repeated irradiation experiments, the formation of prototypic hydrogel sheet dressings loaded with AgNPs was performed in the two-step processing by electron beam irradiation. This latter irradiation platform could support the further up-scale fabrication of AgNP-loaded hydrogel sheet dressings within a shorter processing time. The presence of the AgNPs in the AgNP-loaded hydrogel sheets was confirmed by SEM (Supplementary Materials Figure S1). For the evaluation of the AgNP-loaded hydrogel sheets for their antibacterial efficacy, we selected *S. aureus* and *E. coli* out of the 28 bacteria species [13] commonly found in infected wounds as the representatives of Gram-positive and Gram-negative bacteria. Using the agar diffusion assay (Figure 5), a clear zone formed around each sample with a distinct boundary from the hydrogel’s edge indicating the zone of inhibited bacterial growth. Between the two strains of bacteria, the larger and more prominent clear zones were observed around the AgNP-loaded hydrogels on the *E. coli* plate (Figure 5A). As a control, no clear zone or halo was observed around the hydrogels (*control*) against both *E. coli* and *S. aureus*.

The quantitative analysis of the clear zone and its implication for the antibacterial efficacy of the AgNP-loaded hydrogels was performed by manually measuring the diameter of the clear zone that formed around each sample. The inhibition ratio, which was defined as the diameter of the clear zone normalized by the diameter of the hydrogel, was then calculated in which a ratio above “1” indicated the ability of the AgNP-loaded hydrogel in inhibiting bacterial growth. Table 2 shows that the inhibition ratios against *E. coli* and *S. aureus* increased with the amount of AgNPs loaded in the hydrogels. The total amount of AgNPs on each hydrogel was calculated based on the concentration of the starting AgNO_3_ solution (Table 3 in Section 4 Materials and Methods) at 50 µL per 4 cm^2^ area of hydrogel. For *E. coli*, the amount of AgNPs at the minimal level of 0.25 mg/cm^2^ on the hydrogel could effectively inhibit bacterial growth.

In contrast, it required at least 0.5 mg/cm^2^ of AgNPs on the hydrogels to inhibit the growth of *S. aureus*. Based on these data, *E. coli* was more sensitive to the AgNPs at a lower concentration than *S. aureus*, which was consistent with a previous report [27]. Regarding the antibacterial efficacy against *S. aureus*, the amounts of AgNPs loaded in our hydrogels, 0.5 and 1.0 mg/cm^2^, were within the same range as the commercial dressing coated with silver nanocrystalline at 0.69–1.64 mg/cm^2^ [28]. As we serially varied the amount of AgNPs at a two-fold dilution, the minimal inhibition concentration (MIC) of AgNPs against *S. aureus* and *E. coli* was found to be 0.5 and 0.25 mg/cm^2^, respectively. In terms of the sensitivity of the different bacteria to the AgNPs, our findings also agreed with a previous report that the MIC of AgNPs against *S. aureus* was higher than the level required for *E. coli* [29].

**Figure 6:**
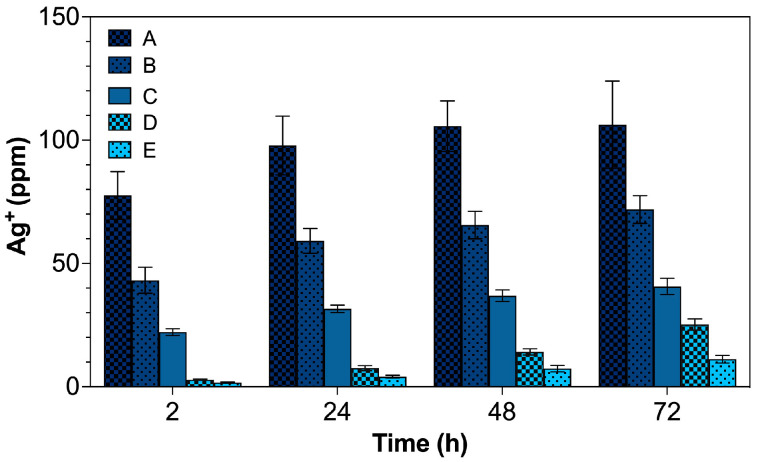
Release of Ag^+^ from the AgNP-embedded hydrogels measured by ICP.

Based on our previous study, the release of AgNPs from the hydrogels was qualitatively confirmed by UV-Vis spectrophotometry [30]. In the present study, the amount of released AgNPs at varied AgNO_3_ loadings was quantitatively measured in the form of ionized Ag^+^ by ICP-MS (Figure 6), which decreased proportionally from treatments A–E. For hydrogels A, B, and C incorporated with AgNPs at 1, 0.5, and 0.25 mg/cm^2^, respectively, the amount of released AgNPs increased sharply by approximately 26–43% between 2 and 24 h. After, the level of the released AgNPs remained relatively unchanged for up to 72 h. Interestingly, for treatments D and E, with a much lower initial AgNO_3_ loading, the released AgNPs continued to rise linearly with the incubation time from 2 up to 72 h. Regarding the AgNP levels of 59 ± 5 ppm for hydrogel B and 32 ± 2 ppm for hydrogel C measured by ICP at 24 h, it can be derived from our experiment that at least ~30 and 60 ppm of AgNPs is required for effective antibacterial action against *S. aureus* and *E. coli*, respectively.

### 2.6. Cytocompatibility

**Figure 7:**
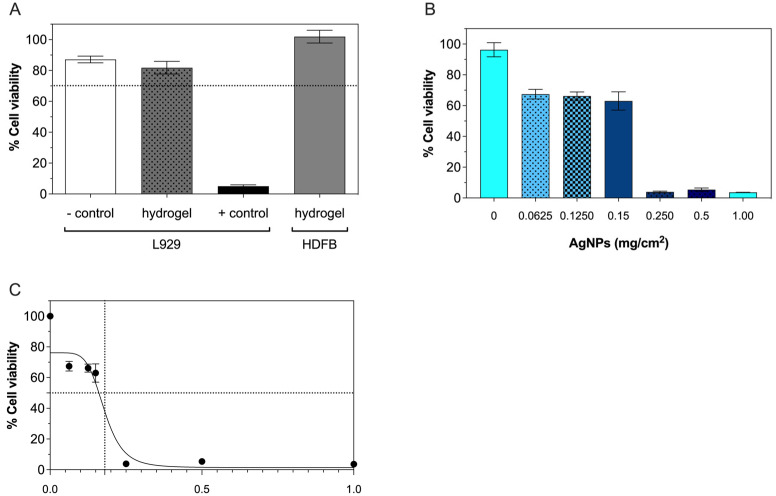
Cytocompatibility evaluation of (**A**) hydrogel sheets with both L929 and HDFB cells and (**B**) AgNP-loaded hydrogel sheets at varied amounts of AgNPs with L929 cells. The dashed line at a cell viability of 70% in (**A**) presents the threshold of non-cytotoxicity. (**C**) Dose–response curve shows the estimation of the IC_50_ by the nonlinear regression analysis with the four-parameter logistic curve fit.

The MTT-based extract test was used to evaluate the cytocompatibility of the nontreated hydrogels and AgNP-loaded hydrogels. For the nontreated hydrogels, a cytotoxicity evaluation was performed in both L929 and HDFB cells compared to positive and negative controls (Figure 7A). After being cultured in extracts derived from hydrogels, the L929 cells showed viability at 80%, similar to the negative control and above the 70% threshold of non-cytotoxicity for medical devices [31]. By contrast, the viability of L929 cells in extract derived from the positive control was below 10%. In addition to L929 cells, the viability of HDFB in extracts derived from the hydrogels was also well above 70%, suggesting that our prototypic hydrogel sheets could be applied for dermal application, such as wound dressings.

For the AgNP-loaded hydrogel sheets, the viability of the L929 cells decreased with the amounts of AgNPs present on the hydrogels (Figure 7B). Over a range of AgNPs from 0.0625 to 0.15 mg/cm^2^, the cell viability gradually decreased from ~96% to 60%. However, the cell viability was sharply reduced to ~5% when the AgNPs increased from 0.25 to 1 mg/cm^2^. To estimate the AgNP concentration required to inhibit the cell viability to 50% (IC_50_), a nonlinear regression analysis was performed on the dose–response curve. Based on the four-parameter logistic curve fit, the calculated IC_50_ value for the AgNPs in hydrogel was 0.18 mg/cm^2^ (Figure 7C).

In this study, we found that AgNPs at the bactericidal level was toxic not only to bacteria but also to mammalian cultured cells. This finding agreed with previous studies that reported the cytotoxic effect of AgNPs in mammalian cells when used at its bactericidal concentration [32,33,34]. Based on these data, it is possible to have both the antibacterial benefit of AgNPs and also the cytotoxic effect of AgNPs on mammalian cells at the same time. Therefore, the antibacterial benefits and potential health risks of AgNPs need to be weighed for further use in clinical application [29]. One promising strategy is to apply AgNPs at the cytocompatible dose in combination with other antibacterial agents to gain a synergistic antibacterial effect. Recently, Ipe et al. [29] successfully demonstrated in both disc diffusion and broth microdilution assays that AgNPs at the cytocompatible dose could enhance the bactericidal effects of the antibiotics against several bacterial species, including antibiotic-resistant bacteria. Therefore, the use of AgNPs in combination with other antibacterial agents, each at their respective cytocompatible thresholds, to gain synergistic antibacterial activity can provide a new approach for the design of wound dressings with antibacterial properties.

## 3. Conclusions

In this study, we demonstrated that the two-step processing by EB irradiation provided an environmentally friendly and convenient platform to fabricate AgNP-loaded hydrogel sheet dressings that can be further developed for wound healing. The basic properties and structure of the crosslinked hydrogels showed that the swelling and *M_c_*, but not the gel fraction and *ρ_x_*, decreased with the increase in the irradiation dose. The optimal doses for the two-step processing were established based on the swelling and stretchability of the hydrogel sheets. While the disc diffusion assay showed that the antibacterial efficacy scaled with the AgNPs loaded in the hydrogels with a stronger effect against *E. coli* than *S. aureus*, the cytotoxicity evaluation showed a sharp decrease in the cell viability. At the minimal inhibition concentration levels, the release of AgNPs from hydrogels followed an immediate increase before remaining relatively unchanged after 24 h. Using this two-step processing approach by EB irradiation, sterilized and antibacterial AgNP-loaded hydrogel sheet dressings were successfully fabricated. The possibility of combining AgNPs with other antibacterial agents still needs to be explored to lower the cytotoxicity effect.

## 4. Materials and Methods

### 4.1. Materials

PVA (MW 89,000–98,000 Da, 99% hydrolysis), sodium carbonate (Na_2_CO_3_), calcium chloride (CaCl_2_), nitric acid (HNO_3_), 3-(4,5-dimethylthiazol-2-yl)-2,5-diphenyl tetrazolium bromide (MTT), isopropanol, ICP-grade silver nitrate (AgNO_3_) standard, fetal bovine serum (FBS), Eagle’s minimal essential medium (MEM), tissue-culture grade water, L-glutamine, and penicillin–streptomycin, Mueller–Hinton agar, and nutrient broth were purchased from Sigma-Aldrich. The L929 mouse fibroblasts (NCTC clone 929) and HDFB (PCS-201-012) were purchased from ATCC. Reagent-grade AgNO_3_ was purchased from Merck. Polyurethane containing 0.1% zinc (RM-A) and high-density polyethylene (RM-C) were purchased from Hatano. *S. aureus* (ATCC 25923) and *E. coli* (ATCC 25922) were purchased from ATCC (ATCC, USA). Deionized water was used to prepare all solutions.

### 4.2. Preparation of the PVA Hydrogels

The PVA powder was dissolved in boiling water at a concentration of 10% (*w*/*v*) and thoroughly mixed by a magnetic stirrer for 1 h. The solution was de-aerated with N_2_ gas, transferred to 8 × 8 cm square Petri dishes or round dishes with a 5.5 cm diameter and then sealed inside plastic bags. The samples were irradiated in a multipurpose gamma irradiator at a dose rate of 4.3 kGy/h to the final doses of 10, 25, 40, 60, and 80 kGy at the Gem Irradiation Center facility, Thailand Institute of Nuclear Technology (Public Organization). As-cast PVA sheets served as a control. For the fabrication of the prototypic hydrogel sheets at a selected dose for further studies, EB irradiation generated by an electron accelerator (MB10–50) with an energy of 10 MeV at 50 kW was applied instead of gamma irradiation.

### 4.3. Hydrogel Characterization

#### 4.3.1. Swelling

Round hydrogel sheets from the 5.5 cm dishes were first rinsed with DI water to remove unreacted PVA. The samples were then fully immersed for 24 h in an aqueous solution containing 142 mmol NaCl and 2.5 mM CaCl_2_, which simulated the discharge at the wound site [26], before being dried at 60 °C to a constant weight. The equilibrium degree of the swelling was calculated as follows:

*EDS (%) = (W_s_ − W_d_)/W_d_* × 100 (4)

where *W_s_* is the swollen weight at 24 h, and *W_d_* is the dried weight. The experiments were performed in quadruplicate.

#### 4.3.2. Gel Fraction

Based on the previously reported protocol [35], the round hydrogel samples were cut in half to an approximately equal weight. One-half of the samples was boiled in DI water for 15 min to extract any loose, non-crosslinked PVA molecules. They were then dried with the remaining half of the samples at 60 °C to a constant weight. The stable gel portion was calculated by the following equation:

*Gel fraction (%) = W_f_/W_o_* × 100 (5)

where *W_f_* is the dried weight of the hydrogels after boiling, and *W_o_* is the dried weight of the starting hydrogels. The lumpy, paste-like samples formed at 10 kGy were first dried at 60 °C to a constant weight (*W_o_*) before being boiled and finally dried to a final constant weight (*W_f_*). The experiments were performed in quadruplicate.

#### 4.3.3. Thermal Analysis

The melting enthalpy and melting point of each sample (approximately 3 mg) was analyzed by differential scanning calorimetry with a DSC 822^e^ (Metler Toledo, Columbus, OH, USA), and an empty crucible served as a reference. The heating cycle was set between 25 and 255 °C with a heating/cooling rate of 10 °C/min under a flow of N_2_ at 60 mL/min. The experiments were performed in triplicate.

#### 4.3.4. Morphology Analysis

The hydrogel sheets, with thicknesses of 5 mm, were first prepared and then frozen at −80 °C in their swollen state overnight. After lyophilization at −49 °C and 0.08 mbar (Christ Alpha 2–4 LD plus, Germany) for 72 h, the samples were immersed in liquid N_2_ for at least 30 min before being quickly fractured. The cut surface was coated with gold palladium and examined by scanning electron microscopy (SEM, Hitachi SU8020, Ibaraki, Japan) at a 1 kV and 10.5 µA setting.

### 4.4. Analysis of the Tensile Properties

The hydrogel sheets were cut into dumbbell shape (ASTM D-1822) with a narrow section 9.53 mm long and 3.18 mm wide. The tensile tests were performed on a texture analyzer (Lloyd LS1, AMETEK, Largo, FL, USA) equipped with 50 N load cell and a crosshead speed set at 50 mm/min. The data were recorded as the load and displacement in quintuplicate per irradiation dose.

### 4.5. Synthesis of the AgNPs by EB Irradiation on the Hydrogel Sheets

The solutions of AgNO_3_ were first prepared at four different concentrations (A–D), as shown in Table 3. Briefly, AgNO_3_ powder was dissolved in DI water and sonicated for 15 min. Then, 50 μL of AgNO_3_ solution was pipetted onto 2 × 2 cm hydrogel sheets that were preformed by EB irradiation at 40 kGy. The hydrogel samples were allowed to fully absorb the AgNO_3_ solution for 3 h in the dark. Next, all of the coated samples were exposed to EB irradiation at 25 kGy to convert the starting AgNO_3_ into AgNPs. The distribution of AgNPs inside the lyophilized AgNP-loaded hydrogel sheet was confirmed by field emission SEM (Hitachi SU5000, Japan) operated at 5 kV using the EDS mode.

**Table 3:** Concentrations of the AgNO_3_ solutions used in the preparation of the AgNP-loaded hydrogels.

Solution	Concentration of AgNO_3_ (mg/mL)
A	80
B	40
C	20
D	10
E	5

### 4.6. Antibacterial Properties

The antibacterial activities of the AgNPs loaded hydrogel sheets were first evaluated by the agar diffusion method against *S. aureus* and *E. coli.* The concentration of the bacterial suspension was determined by absorbance at 600 nm with an optical density (OD) of 0.2, corresponding to 10^7^ colony forming units (CFUs) per milliliter. The hydrogel samples were cut by an 8 mm diameter puncher under aseptic conditions and then placed on the agar plates inoculated with 10^6^ CFU of either *S. aureus* or *E. coli*. After 20 h incubation at 35 °C, the clear areas that formed around the samples were recorded and measured in three directions. The experiments were performed in triplicate.

### 4.7. In Vitro Cytotoxicity Test

The cytotoxicity was determined by the MTT-based extract test. The hydrogel sheets, AgNP-loaded hydrogel sheets, and negative and positive controls were first extracted in MEM supplemented with 100 IU mL^−1^ penicillin streptomycin, 4 mM L-glutamine, and 10% (*v*/*v*) FBS (complete media) inside 15 mL centrifuge tubes for 24 h. Complete media in tubes without sample served as the blank control. The L929 and HDFB cells were seeded in 96-tissue culture plates at a density of 1 × 10^4^ cells per well in complete MEM and incubated in a humidified incubator at 5% CO_2_ and 37 °C for 24 h before being refreshed with 100 µL of extracts and further incubated for 24 h. The extracts were then removed, and 50 µL of freshly prepared MTT solution in MEM without phenol red at a concentration of 10% (*w*/*v*) was added to each well. After 3 h of incubation, the resulting formazan crystals formed inside the cells were dissolved in 50 µL of isopropanol. The absorbance of the purple solution was measured with a microplate reader (SpectraMax M3, Molecular Devices, San Jose, CA, USA) at 570 nm. The cell viability was determined based on the absorbance ratio of each sample over the blank control. The experiments were performed in quadruplicate.

### 4.8. Inductively Couple Plasma-Mass Spectrometry Analysis

The release of AgNPs was quantitated by ICP-MS. The AgNP-loaded hydrogels were digested in 6 mL of 1% (*v*/*v*) HNO_3_ for 4 h to covert silver to Ag^+^. To construct the calibration curve, Ag solution in 2% (*v*/*v*) HNO_3_ (ICP-grade) was diluted to 5, 25, 50, 75, 100, and 250 µg/L. The samples were analyzed on an ICP-MS (Agilent 7900, Agilent Technologies Japan, Ltd., Tokyo, Japan).

### 4.9. Test of Sterility

The hydrogel sheets without AgNPs (square sheets: 8 × 8 cm and 2 × 2 cm; circular sheets: 5.5 cm diameter) were immersed in 250 mL of Modified Letheen Broth and incubated at 30 °C for 14 days. Any occurrence of turbidity, sediment, flocculation, and color changed indicated the growth of microorganisms in the broth and would be interpreted as nonsterile. If the broth remained clear after 14 days, the pour plate method was further performed by incubating the plate count agar containing 1 mL of broth at 30 °C for 4 days. The absence of a microorganism colony on the plate count agar would be interpreted as sterile. Finally, the suitability test was performed by inoculation of 10–100 CFUs of *Escherichia coli* in the medium containing the product. The suitability test was qualified if the presence of microorganisms was observed.

### 4.10. Statistical Analysis

The data are presented as the mean ± standard deviation. All statistical analyses were performed using GraphPad Prism version 9.2.0 (GraphPad Software, San Diego, CA, USA). The normality of the data distribution was performed using the Sharpiro–Wilk test. For the EDS, tensile strength, Young’s modulus, and elongation at break data, a comparison between the two means was carried out using the unpaired Student’s *t*-test. For the gel fraction data, the Mann–Whitney test was used for comparison between two means. The nonlinear regression analysis was performed on the dose–response curve using four-parameter logistic curve fitting to estimate the IC_50_. The *p*-value < 0.05 was considered statistically significant.

## Figures and Tables

**Table 1:** Gel fraction, equilibrium degree of swelling (EDS), and network parameters (*M_c_*, *ρ_x_*, and *ε*) of crosslinked PVA hydrogels as a function of irradiation doses. Each value of the EDS and gel fraction data represents the mean ± SD (n = 4).

Dose (kGy)	Gel Fraction (%)	EDS (%)	*M_c_* (g/mol)	*ρ_x_* (10^−4^ mol/cm^3^)	*ε* (Å)
10	25.1 ± 16.1	-	-	-	-
25	86.6 ± 5.1	2554 ± 294	16,240	0.8	358
40	93.5 ± 2.9	1348 ± 222	6770	1.9	173
60	92.0 ± 7.0	1311 ± 133	6250	2.0	163
80	93.8 ± 1.0	892 ± 17	3300	3.9	130

Note: The EDS at 10 kGy was not obtainable due to the difficulties in the sample handling.

**Table 2:** The inhibition ratios of the AgNP-loaded hydrogels against *E. coli* and *S. aureus*. The data are represented by the mean ± standard deviation from 3 replicates, each measured in 3 directions. The dash indicates the absence of a clear zone.

Sample	Total AgNPs (mg/cm^2^)	Inhibition Ratio	
		*E. coli*	*S. aureus*
Hydrogel A	1.0	1.78 ± 0.09	1.50 ± 0.01
Hydrogel B	0.5	1.66 ± 0.01	1.26 ± 0.02
Hydrogel C	0.25	1.50 ± 0.17	-
Hydrogel D	0.125	-	-
Hydrogel E	0.0625	-	-

## Data Availability

All data are reported in the manuscript.

## Supplementary Materials

The following supporting information can be downloaded at: https://www.mdpi.com/2310-2861/9/2/80/s1, Figure S1: SEM analysis of the AgNP-loaded hydrogel. (**A**) The presence of AgNP clusters as pointed by arrows inside the hydrogel. (**B**) The analysis of the clusters in (**A**) using EDS. (**C**)–(**H**) The corresponding EDS spectra showing Ag Lα_1_ signals in the clusters specified in (**B**).
