# Correction: Chiangnoon et al. Antibacterial Hydrogel Sheet Dressings Composed of Poly(vinyl alcohol) and Silver Nanoparticles by Electron Beam Irradiation. *Gels* 2023, *9*, 80

**DOI:** 10.3390/gels9100778

**Published:** 2023-09-25

**Authors:** Rattanakorn Chiangnoon, Pennapa Karawak, Jarurattana Eamsiri, Sasikarn Nuchdang, Nuatawan Thamrongsiripak, Naruemon Neramitmansook, Siwanut Pummarin, Pimchanok Pimton, Kewalee Nilgumhang, Pimpon Uttayarat

**Affiliations:** 1 Nuclear Technology Research and Development Center, Thailand Institute of Nuclear Technology (Public Organization), Ongkarak, Nakhon Nayok 26120, Thailand; rattanakorn@tint.or.th (R.C.); pennapa@tint.or.th (P.K.); jaruratana@tint.or.th (J.E.); sasikarn@tint.or.th (S.N.); 2 Irradiation Center, Thailand Institute of Nuclear Technology (Public Organization), Ongkarak, Nakhon Nayok 26120, Thailand; nuatawan@tint.or.th (N.T.); naruemon@tint.or.th (N.N.); 3 Department of Biology, School of Science, Walailak University, Nakhon Si Thammarat 80160, Thailand; p.siwanut@gmail.com (S.P.); pimchanok.pi@mail.wu.ac.th (P.P.); 4 Program in Medical Sciences, Faculty of Medicine, Chulalongkorn University, Bangkok 10330, Thailand; 5 Functional Materials and Nanotechnology Center of Excellence, Walailak University, Nakhon Si Thammarat 80160, Thailand; 6 Advanced Engineering and Nuclear Technology Center, Thailand Institute of Nuclear Technology (Public Organization), Ongkarak, Nakhon Nayok 26120, Thailand; kewalee@tint.or.th

## Error in Figure

In the original publication [1] there was a mistake in Figure 6 regarding the amount of AgNPs being released from hydrogels. Because the calculation of released AgNPs was based on the sampling volume of 30 μL rather than the total volume of 2 mL that each AgNP-loaded hydrogel had been immersed in, the amount of released AgNPs was underestimated. After correction, the total amount of released AgNPs was at the ppm level as shown below in the corrected Figure 6.


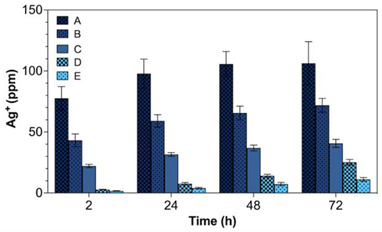


Due to this correction in Figure 6, the reported values of the released AgNPs in the original publication [1] from hydrogels B and C that correspond to the MIC levels are 59 ± 5 ppm and 32 ± 2 ppm, respectively, as measured by ICP at 24 h. In addition, the amount of released AgNPs increased sharply by 26–43% between 2 and 24 h. Therefore, it can be derived from our experiment that at least 30 and 60 ppm of AgNPs are required for the effective antibacterial action against *E. coli* and *S. aureus*, respectively.

The authors state that the scientific conclusions are unaffected. This correction was approved by the Academic Editor. The original publication has also been updated.
