# Supplementary material for: Antibacterial Hydrogel Sheet Dressings Composed of Poly(vinyl alcohol) and Silver Nanoparticles by Electron Beam Irradiation
Source: Gels. 2023 Jan 18;9(2):80. doi: 10.3390/gels9020080 (PMC9957089; doi:10.3390/gels9020080)
Supplement: Supplementary file 1 [file gels-09-00080-s001.zip › gels-2101549-supplementary.pdf]

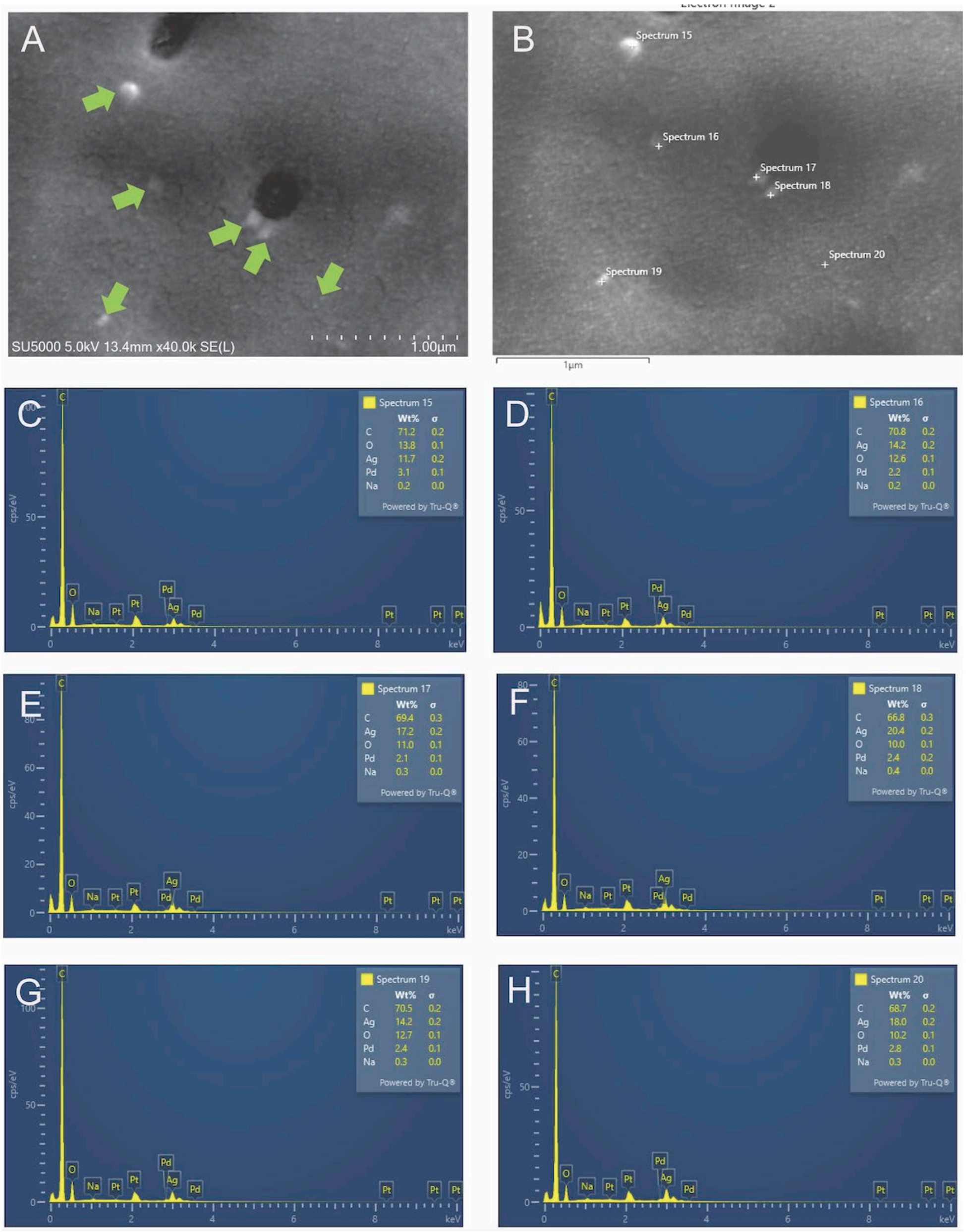

Supplementary Figure S1. SEM analysis of AgNP-loaded hydrogel. (**A**) The presence of AgNP clusters as pointed by arrows inside hydrogel. (**B**) The analysis of clusters in (**A**) using EDS. (**C**)–(**H**) The corresponding EDS spectra showing Ag Lα_1 signals in clusters specified in (**B**).
